# Assessment of Postharvest Practices of Tuna Sold at the Honiara Fish Market in the Solomon Islands

**DOI:** 10.1155/2023/6594017

**Published:** 2023-08-19

**Authors:** Madeline Kili Solo, Jimaima Lako, Francis Mani, Gilianne Brodie

**Affiliations:** ^1^Department of Fisheries Studies, Solomon Islands National University, Solomon Islands; ^2^School of Applied Sciences, Fiji National University, Fiji; ^3^School of Agriculture, Geography, Environment, Oceans & Natural Resources, The University of the South Pacific, Fiji; ^4^Institute of Applied Sciences, The University of the South Pacific, Fiji

## Abstract

The study is aimed at assessing the impacts of postharvest handling practices on the quality and safety of tuna sold at the Honiara Fish Market (HFM), Solomon Islands. Two major approaches were adopted: (1) face-to-face interviews of 60 participants using questionnaires and physical observations of the supply chains and postharvest handling practices and (2) determination of time-temperature, quality index, histamine, and microbial load of tuna and contact surfaces. Sampling was conducted on both the wet season (WS) and dry season (DS), of which 36 samples from both batches of fresh tuna (FT) and brined tuna (BT) were analyzed. Three critical control points (CCPs) were identified in the supply chains of both FT and BT, where samples were obtained for scientific analyses. The average body temperature for WS tuna exposed for 9-10 h with low or no ice after catch was 3°C for FT and 15°C for BT, while DS samples were 26°C and 31°C for FT and BT, respectively. The quality index (QI) for WS showed a significant difference (*P* < 0.05) at 0 for FT and 8 for BT, while both DS showed a significant increase at 16 for BT and 5 for FT. Histamine levels for all the samples increased across the three CCPs, however with levels <50 mg/L, while microbial load for both seasons and for both samples were within the required specifications. However, contact surfaces for both seasons revealed high levels of microbial contamination. This study reveals that poor handling practices along the tuna supply chains of fish sold at the HFM were observed; however, all the tuna was safe for consumption when cooked properly.

## 1. Introduction

The Solomon Islands is situated in the Western Pacific and has abundant marine resources, with tuna the most common fish species caught, traded, and consumed, which contributes to the development of individuals, families, and the country as a whole [1]. The tuna industry in the Solomon Islands not only contributes to the national and domestic economy but has also been part of their cultural significance [2]. The aim of the study was to assess the impacts of postharvest handling practices on the quality and safety of tuna sold at the Honiara Fish Market (HFM) in the Solomon Islands beginning from the time of capture up to auction. This assessment involved physical, sensory, chemical, and microbiological analyses.

Tuna is one of the fish species that is highly valued with rapid increase in popularity in some countries around the world [3]. Its high market demand may be due to the good flavor, high nutritional value, and high lipid content of the various tuna species [4]. Nutritionally, tuna contains about 5.11% eicosapentaenoic (EPA, 20 : 5n3) and 23.9% docosahexaenoic (DHA, 22 : 6n-3) acids, which are associated with the reduction of risks related to heart diseases [5].

Despite the high nutritional value of tuna, they are still perishable due to poor handling practices like many other scombroid fish species, which results in the development of high histamine levels that can cause Histamine Fish Poisoning (HFP). HFP is the most common fish-related intoxication [6] associated with tuna and also other scombroid fish such as mahi-mahi and mackerel [7]. The formation of histamine in the tuna flesh is attributed to a range of microorganisms, such as *Hafnia alvei*, *Morganella morganii*, *Morganella psychrotolerants*, *Photobacterium phosphoreum*, and *Klebsiella pneumoniae* [8]. The safety requirements of HFP in tuna and other scombroid fish were specified by the European Commission in the Regulation (EC) no. 2073/2005 and the subsequent modifications of Commission Regulation (EC) no. 2073/2005 [9]. Other significant concerns of tuna consumptions were made by the Health and Food Safety Commission in 2016 about the treatment of canned tuna with the use of vegetable extracts containing high levels of nitrates/nitrites [10]. Similarly, other reports of scombroid fish poisoning occurred in Spain, Puglia and Basilicata regions (south part of Italy) were received after consumption of tuna-based meals [11]. When tuna samples that were used in the meal preparations were analyzed in the laboratory by the competent authorities, they were found to be noncompliant to the histamine limits provided by the EC.

Histamine is a biogenic amine (BA) that has a great sanitary and technological importance both in relation to anaphylactic poisoning episodes and as a freshness indicator [12]. Other biogenic amines include tyramine, cadaverine, putrescine, and other related metabolites that are produced from the decarboxylation of amino acids. Significantly high amounts of biogenic amines are produced during processing and storage of seafood due to microbial contamination as a result of poor handling practices and inadequate storage conditions [13]. Biogenic amine index (BAI) is a sensitive indicator of meat freshness and quality of which the limit of fish acceptability for quality index is 10. Any biogenic amine index value exceeding 10 is regarded as loss in quality [14]. Hence, due to the perishable nature of seafood including tuna and its significant health concerns, proper postharvest management especially immediate refrigeration or the use of ice to sustain its freshness, quality and safety standards [15]. This means that preparation, handling practices and storage of fish both at domestic and commercial levels are important processes that need to be monitored to control bacterial growth and deterioration [16].

Improper handling practices and inadequate or poor infrastructural facilities may have direct impacts on the quality of fish, which leads to significant postharvest losses [17]. There are three types of postharvest losses, namely, physical, quality, and market losses, respectively. Physical loss is when fish deteriorate and are not able to be sold or used after capture or when landed. It is either thrown away accidentally, voluntarily or as authorized. Physical loss can be caused by theft, insects eating fish, or by bird or animal predation. Quality loss in fish on the other hand is when fish flesh goes through chemical changes, resulting in spoilage or physical damage that contributes to quality deterioration. Such fish is often sold at a lower price compared to the ones that would achieve the best quality. Likewise, market loss is when fishers sell their fish at a lower price, below their expectations, despite its good quality [18]. This mainly occurs due to unfair market practices and pricing [19]. Minimizing all these three types of postharvest losses, may improve and increase quality and quantity of fish available to consumers, which may in turn improve fish supply, food security, and the sustainability of the fishery industry.

Globally, fish losses due to poor postharvest handling are estimated to be ten to twelve million per year, accounting to around 10% to 30% of total production [20]. This is supported by [19] who argued that not all the fish that are harvested reach the dinner table and that 70% of fish loss occur along the production chain. Such fish losses reduce fish supplies at both domestic and export markets. In many developing countries, post-harvest losses of fish due to poor postharvest handling practices are one of the many challenges faced mostly by artisanal or small-scale fisheries of which Solomon Islands is no exception.

## 2. Materials and Methods

Various methods were adopted to gather appropriate information for the supply chains and postharvest fish handling practices in the Solomon Islands. These include the use of questionnaires, in-depth interviews, determinations of histamine, microbial load, and time and temperature, as well as observations, while the supply chains were walked through.

### 2.1. Chemicals

The reagent-grade Kikkoman Histamine Test Calorimetric Assay Kit (http://www/hyserve.de/files/aHistamine%20Flyerpdf) that was used for histamine determination was brought from Altimed Australia Pty Ltd., while the laboratory-grade Peptone Saline Water (PSW) and the Plate Count Agar (PCA) that were used for microbial analyses were bought from Thermo Fisher Scientific.

### 2.2. Face-to-Face Interviews with Participants

A total of 60 participants were selected, of whom 30 were artisanal fishers (AFs) and 30 were fish retailers (FRs). These participants were randomly selected from the list of 300 registered members of the Artisanal Fishermen Association of Solomon Islands (AFASI). Face-to-face interviews were carried out together with general observations along the supply chain.

### 2.3. Sampling of Tuna

Samples of the two sets of tuna were collected on two separate seasons: wet season (WS) (August to September) and dry season (DS) (December to January). These samples were identified through the development of 3 critical control points (CCPs) for both fresh tuna (FT) and brined tuna (BT) supply chains. The approach adopted for identifying the CCPs was based on the Hazard Analysis Critical Control Points (HACCP), a preventive method of reducing risks in order to produce safe food products [21]. Hence, the sampling points used for this study were the likely areas where hazards would have occurred when good handling practices are not practiced.

### 2.4. Quality and Safety Assessments of Tuna

Tuna samples collected from the 3 CCPs were first assessed for quality index score using quality index (QI) methods in the Solomon Islands before these were wrapped with sterile glad wrap (party time cling wrap), labelled and stored in the freezer at -80°C, ready for air freighted to Fiji for further analyses.

Further analyses for time and temperature (TT), histamine, and microbial analyses were performed on both tuna samples, FT and BT, and from both the seasons WS and DS and their respective contact surfaces.

### 2.5. Quality Index (QI) Score

The QI scores of all tuna samples received at each CCP were determined using the QI score worksheet criteria of [22–24] while conducting organoleptic assessments.

### 2.6. Time and Temperature (TT) Measurements

The TT of tuna that was sampled from each CCP for both fresh and brined tuna on both WS and DS was measured using the ibutton temperature data logger (ITDL) (DS 1921G) that was purchased from Maxim Integrated Products (Sunny Vale, CA, USA). The data obtained from the ITDL was extracted, cleaned, and recorded into an Excel worksheet.

### 2.7. Histamine Determination

Histamine was determined using the test kit procedure of Sato [25]. The analyses were conducted with a Kikkoman Histamine Test Colorimetric assay kit (http://www/hyserve.de/files/aHistamine%20Flyerpdf) brought from Altimed Australia Pty Limited. Prior to using the Histamine Kit Photometer, it was calibrated following the instructions extracted from KYORITSU CHEMICAL–CHECK Lab., Corp (http://kyoritsu-lab.co.jp). Pretested and validation of the method were done for histamine analysis using tuna samples brought from the same study site. Samples were carried out in triplicates, and the same samples were sent to The Institute of Applied Sciences (IAS), the University of the South Pacific, Fiji for High-Performance Liquid Chromatography (HPLC) analyses as interlaboratory comparison and quality assurance. The limit of quantification (LOQ) for the histamine kit was 10 mg/L.

In brief, approximately 10 g of tuna flesh was obtained from the nape area as per Baranowski et al. and Chamberlain's [26, 27] methods. From a 10 g homogenized sample, 1 g was transferred to a heat resistance test tube which was treated with 24 ml of buffered EDTA-2 Na solution pH 8.0, boiled for 20 minutes then cooled in ice prior to filtering. The liquid phase was used for histamine analysis using UV Spectrophotometer, at absorbance 470 nm, Kyoritsu Chemical; Corporation, Model ABS-B470.

### 2.8. Microbial Analyses

#### 2.8.1. Tuna Flesh Samples

The same whole tuna obtained for histamine samples was also sampled and prepared aseptically by homogenizing 10 g with 90 mL (1 : 9 *w*/*v*) of sterile buffered peptone water diluents for 1 minute using a stomacher. The serial decimal dilutions from 10^−1^ to 10^−4^ of each homogenate were prepared with saline water (0.85% NaCl) for microbial analysis [28], and the results were expressed as cfu/mL.

#### 2.8.2. Tuna Contact Surfaces

The contact surfaces of where whole tuna were sold were swabbed twice per day for 3 days in a week at 6 am before auction and 5 : 30 pm after auction. The microbial analytical procedures for the European Parliament [29] were adopted for this research with minor modifications. Swabs were taken from the center of the contact surfaces, covering an area of 10 × 10 cm^2^ using a sterile 3M™ (Themofisher, NZ) environmental quick swabs. The swabs were placed in a 9 mL peptone saline water (PSW), rotated the bottle thoroughly for few seconds prior to placing in a small foam insulating box (IB) and transported to the National Public Health Laboratory (NPHL) in Honiara, Solomon Islands for further analysis. A 1 mL of 10^−1^ dilution was transferred into a sterile petri dish with the same procedure, repeated for dilution 10^−2^ to 10^−4^ after which 16 ml of plate count agar (PCA) (Merck, KgaA) and aseptically pipetted over the inoculum. Samples were incubated at 35°C for 48 h. Colonies were then counted using APHA [28] method with results expressed as cfu/ml. Analyses were done in duplicate.

### 2.9. Statistical Analyses

Data for both tuna flesh (FT and BT) and contact surfaces of both seasons (WS and DS) were analyzed using SPSS [30]. Distribution for all data collected was tested for normality using the Shapiro-Wilk test, and nonparametric test was used as described by Lawless and Heymann [31]. The Man-Whitney *U* Test was conducted to test for significant difference among samples (*P* > 0.05). The Spearman's rank correlation coefficient (Spearman's rho) was used to determine the correlations between the following parameters: TT, QI score, microbial, and histamine data.

## 3. Results

### 3.1. Tuna Supply Chains (Fresh and Brined)

Two separate supply chains of tuna, fresh and brined, were developed for artisanal fishers and commercial fishers, respectively, also capturing both wet and dry seasons.

#### 3.1.1. Fresh Tuna (FT)

**Figure 1:**
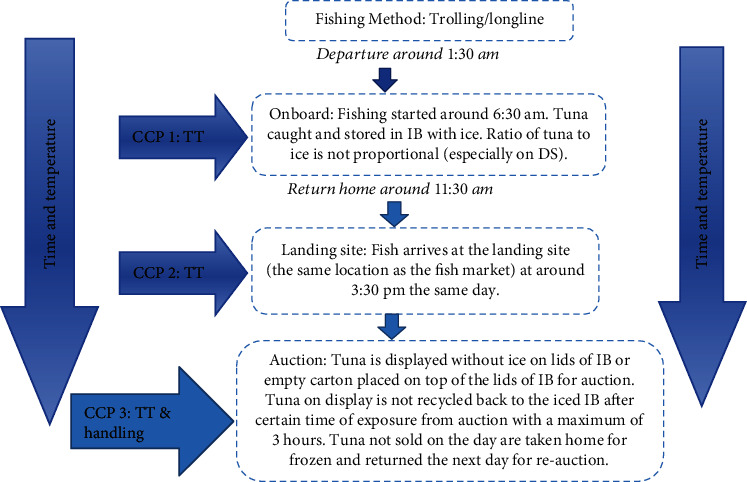
Supply chain for fresh tuna.

A total of three CCPs, onboard (CCP 1), at the landing site (CCP 2) and auction time (CCP 3), were identified for the FT that were harvested and handled by the AFs themselves (Figure 1). The identification of CCPs along the supply chain was very important in the control of any significant food safety hazard that was likely to cause adverse health effect on the tuna products when consumed. The postharvest handling practices for FT that were harvested either through long-line fishing or trolling methods appeared to be similar for both dry and wet seasons, as demonstrated in Figure 1.

#### 3.1.2. Brined Tuna

**Figure 2:**
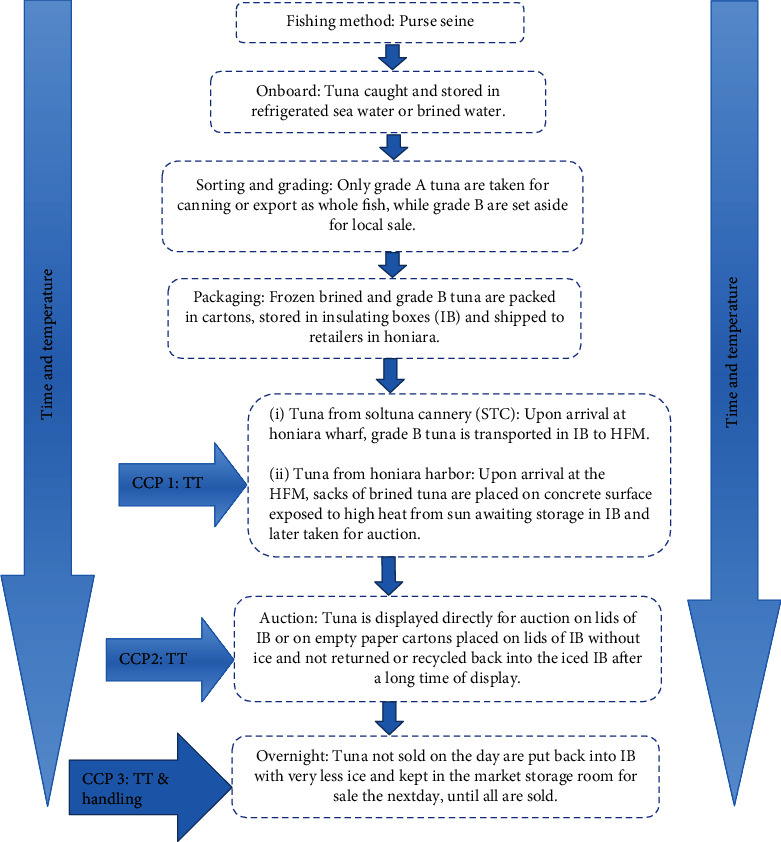
Supply chain for brine tuna.

As shown in Figure 2, the supply chain of brined tuna is caught by commercial fishers. Upon arrival on shores, tuna is graded either as A or B. Grade A tuna is high-quality tuna that meets the export market criteria. These are accepted and transported to the cannery for further processing for export. Grade B tuna on the other hand is usually undersized, weighing ≤1.0 kg and having bruises on the bodies, which is also referred to as bycatch. These bycatches are brined, also called “salt fish,” a term commonly used for the preservation method used onboard fishing vessels. These grade B tunas are usually rejected for export and that are sold to retailers in Honiara. They are usually packed in cartons, placed in insulating boxes, and shipped to Honiara. These were used for the current study.

### 3.2. Histamine Determination and Analyses of Other Related Factors

**Figure 3:**
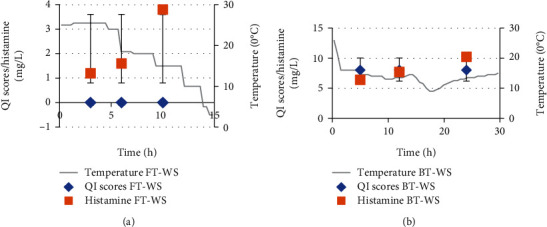
(a) Time and temperature, quality index scores, and histamine levels for WS fresh tuna. (b) Time and temperature, quality index scores, and histamine levels for WS brine tuna.

**Figure 4:**
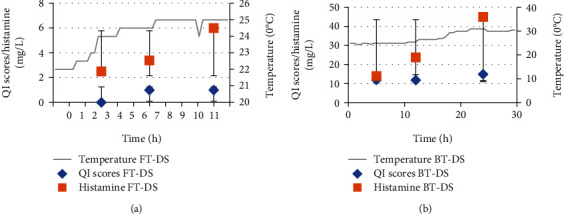
(a) Time and temperature, Quality index scores and histamine levels for DS fresh tuna. (b) Time and temperature, Quality index scores and histamine levels for DS brine tuna analyzed.

**Figure 5:**
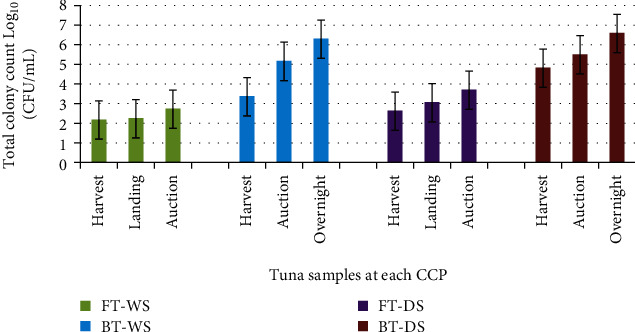
Comparison of the microbial levels of fresh and brined tuna on both wet and dry seasons.

**Figure 6:**
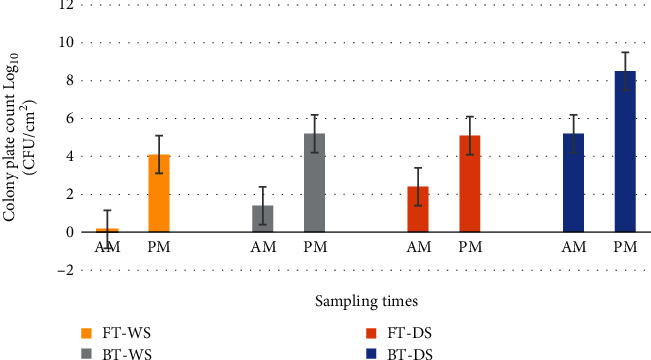
Microbial levels for contact surfaces of fresh and brined tuna samples on both wet and dry seasons.

The average body temperature for WS tuna exposed for 9-10 h with low or no ice after catch was 3°C for FT and 15°C for BT, while DS samples were 26°C and 31°C for FT and BT, respectively. Development of histamine is usually associated with increasing temperature above 4°C which can also be accompanied by increasing bacterial growth [32]. Figures 3(a) and 3(b) and Figures 4(a) and 4(b) show the histamine levels and its related factors including TT and QI for both samples of dry and wet season tuna, respectively, while microbial analyses of flesh and contact surfaces for tuna samples obtained for wet and dry seasons are shown in Figures 5 and 6, respectively. Our data shows higher levels of histamine in DS of which FT recorded 6 mg/L and BT was 45.1 mg/L compared to WS samples which recorded 3.8 mg/L for FT and 10 mg/L for BT; however the levels were below 50 mg/L; the Food and Drug Administration (FDA) [21] and 100 mg/L the European Union (EU) permitted levels European Parliament [29]. Significant differences (*P* > 0.05) were observed between the FT and the BT samples obtained for both seasons.

### 3.3. Microbial Analyses

#### 3.3.1. Tuna Flesh Samples

Microbial levels of both fresh and brined tuna for wet and dry seasons at different CCPs are shown in Figure 5. It shows an increasing trend of microbial levels in both fresh and brined tuna flesh are transported from CCP 1 to 3. For example, the data shows that FT samples for WS increase in microbes from harvest site at CCP1 to the auction site at CCP 3, with a total colony count ranging from 2.1 cfu/ml to 2.7 cfu/mL, respectively, while BT of the same season has the total colony counts of 3.3 cfu/mL at CCP1 (harvest site) and 6.3 cfu/mL at CCP 3 (overnight). No significant differences in the microbial levels (*P* > 0.05) were observed between CCP1 (harvest site) and CCP 2 (landing site), but significant differences (*P* < 0.05) were observed between CCP 1 (harvest site) and CCP3 (overnight).

#### 3.3.2. Tuna Contact Surfaces

As expected, microbial analyses for the contact surfaces of fresh and brined tuna samples also showed increasing trends of microbial level for the afternoon samples compared to the morning samples (Figure 6). The data revealed that the contact surfaces of fresh and brined tuna for WS showed low levels of total colony counts in the morning at 0.15 log cfu/cm^2^ compared to the afternoon at 4.1 log cfu/cm^2^. On the other hand, microbial levels for BT contact surfaces of the same seasons also showed increasing trends with a total colony count of 5.2 log cfu/cm^2^ in the afternoon compared to 1.4 log cfu/cm^2^ in the morning as also shown in Figure 6.

Similar trends were also observed (Figure 6) for DS contact surfaces of fresh and brined tuna. There were low levels of total colony counts for the morning samples at 2.4 log cfu/cm^2^ compared to the afternoon samples at 5.1 log cfu/cm^2^. Likewise, contact surfaces for BT also showed increasing trend for the afternoon samples at 8.5 log cfu/cm^2^ compared to 5.2 log cfu/cm^2^ for the morning samples. Significant differences (*P* < 0.05) were observed among contact surfaces for both fresh and brined tuna samples on both wet and dry seasons.

### 3.4. Correlations between Time-Temperature (TT), Quality Index (QI), and Histamine

Correlations between the TT, QI, and histamine data showed strong correlations between time-temperature and histamine (*r* = 0.91), quality index and histamine (*r* = 0.85), and quality index and time-temperature (*r* = 0.80) (Table 1). Positive correlations were observed between time-temperature and microbial levels (*r* = 0.70), histamine and microbial levels (*r* = 0.62), and quality index and microbial levels (*r* = 0.60). These results strongly confirm the relationships between the increasing histamine levels and exposure of tuna to high temperature, which may be due to the breaking of the cold supply chains related to poor postharvest handling practices by both AFs and FRs.

## 4. Discussion

### 4.1. Fresh Tuna Supply Chain

Based on the information gathered from the in-depth interviews, it appears that poor postharvest handling practices have been practiced as evident in Figure 1. Time-temperature were the major factors that determined the quality and safety of tuna along the supply chain. The detailed data revealed that the majority of artisanal fishers' harvest tuna overnight, with approximately 5-6 hours of fishing (e.g., depart home around 1 : 30 am, harvest from 6 : 30 am, and make their return from 11 : 30 am). It was observed that fish harvested during these hours are not usually gutted, as fishers were rushing to sell their catch at the Honiara fish market to have sufficient time to sell. Hence, fishers usually sell around 3 : 30 pm at the fish market. Given the hot summer weather in the Solomon Islands above 28°C during daytime, melting of ice is expected to be at a faster rate, demanding the use of more ice with good insulating ice boxes.

Depending on fish load and weather, fishing trips undertaken by AFs are usually of long duration including selling at the market, which takes approximately 9 to 10 h in total. The problem with long duration of fishing trips by AFs is that they do not carry sufficient amount of ice to retain the cold supply chain with respect to the high volume of fish caught, in order to maintain freshness and quality. The use of sufficient ice in chilling fish at harvest point is quite an extremely effective means of retaining the freshness of fish while reducing spoilage of fish as argued by Akintola and Bakare [33]. However, based on interviews and observations, our data revealed that the ratio of ice to tuna harvested appeared to be disproportionate. There has been high amount of tuna caught to low volume of ice ratio used, resulted in all ice being melted out upon arrival at the fish market. This often occurs with the tuna harvested during dry season especially due to the high temperature received from strong sunlight. The high rate of the melting of the ice was also observed when there is higher catch load of tuna, which apparently accumulated high body temperatures during storage. This appears to support Burns [34] theory that tuna are distinctive among all fish as they have body temperatures above the surrounding seawater temperature.

It was also noted that upon arrival at the market, tuna is displayed on lids of insulating boxes (IB) or paper cartons for auction without ice and without being recycled back for cooling in the iced IB after certain time of exposure especially when displayed for auctioned for a maximum period of 3 h. Fish is a perishable product and hence has to be stored at low temperatures using ice to retain freshness and avoid spoilage. The current handling practices may have contributed to rapid spoilage as observed by Chamberlain [27] that the humid temperature experience in the Pacific provides the optimal temperature for microbial growth that is responsible for rapid spoiling of fish within 12-20 h of poor handling practices.

### 4.2. Brined Tuna Supply Chain

It was observed that the quality of the tuna caught during DS was mostly low. These tunas when brought for auction do not comply with the EU food safety regulation [9], as these are usually placed directly on dirty concrete floors without ice and are simultaneously exposed to high heat for at least more than 3 h awaiting storage in IB and prior to auction. Placing fish directly on dirty concrete without ice exposes the fish directly to the sun with high environmental temperature above 28°C that violates and breaks the cold supply chain regulation. This puts the brined tuna into high risk of microbial contamination with expected high rate of fish spoilage. However, the use of high-salt concentration in the brine solution appear to preserve the fish and hence reduce spoilage. Several studies by Whittle et al. [35] and Olafsdottir et al. [36] suggest that spoilage process of fish depends on various factors including fish species, handling practices, and storage conditions along the supply chain. The control of cold temperature along the supply chain is essential to ensure that the freshness of the fish is maintained. It was observed that during the auction of brined tuna in Honiara, the ice that was cooling the fish melted away without being replenished, as these tunas were placed directly on the lids of IB or paper cartons without ice and without being recycled back into IB after a long duration of exposure. This similarly demonstrates the noncompliance and breaking of the cold chain requirements which result in the deterioration of the quality of fish, contributing to the reduction of its shelf life [37, 38].

It was also observed that all unsold tuna were put back into the IB with considerably less ice at the end of the day and were brought to be sold the next day, until all brined tuna were sold out. Such practices may be a noncompliance to the food safety regulation [39], which needs further investigation for corrective actions. This may require the relevant authorities to take some stringent measures on such practices and to also provide easier access to ice and other storage facilities for the FRs to properly store their fish overnight to avoid contamination, deterioration, and spoilage.

### 4.3. Relationships between Temperature, Histamine and Quality Index (QI)

Development of histamine is associated with high temperature and bacterial contamination [32].  Figure 3(a) shows that FT-WS had a decreasing trend in temperature that reached 3°C after more than 10 h of fishing. This low temperature-storage correlated with the lower QI scores of 0 (the lower the QI, the fresher the fish) and low histamine levels at 3.8 mg/L (0.85), which reveal that tuna still have a good level of freshness. Retaining lower temperatures of lower catch load during wet season released less heat from tuna [34]. On the other hand, BT of the same season (Figure 3(b)) showed much higher temperatures compared to FT with temperature reaching 15°C. The increase in temperature is also associated with higher QI scores of 8 (the higher the QI score, the lower the freshness) and histamine level at 10.2 mg/L (Figure 3(b)). It was observed that the ratio of ice to fish for BT-WS was very less compared to the quantity of tuna caught. The data confirms the importance of using correct ice ratio to fish during storage. The use of ice for storage of fish helps to preserve and extends the shelf life of the fish by lowering the temperature [40]. A similar study conducted in Fiji by Lako et al., [41] confirmed that histamine levels of Giant Trevally (*Carax ignobilis*) stored at 28°C for 15 h postharvest increased to 193.2 mg/L, which exceeded the EU-permitted levels of 10 mg/100 g (100 mg/L) [32]. This confirms the importance of the continuous use of the right ratio of ice to maintain the cold chain along the supply chain. If the temperature of the fish is decreased close to 0°C, the metabolic activity in the fish is reduced or stopped. Generally, in hotter regions like the Solomon Islands, a rule of thumb is often used. i.e., for every 1.0 kg of fish, 1.0 kg of ice are used. It is important to note that during the peak fishing periods, there is usually an increase in the demand of ice as experienced with the DS tuna in the current study.

Further results for DS tuna revealed that FT temperature increased to 25°C after more than 10 h of fishing (Figure 4(a)). As mentioned above, there are strong correlations between high temperature and low QI scores (1) at CCPs 2 and 3 with increased histamine level (6 mg/L) at CCP 3. Although there is increase in temperature for FT for this DS, the levels of histamine increased only to a small degree. According to Allen [42] and Omura et al. [43], the population of histamine-forming bacteria due to increase in temperature does not always associate with high histamine levels in fish samples but could also be due to either the presence of histamine-producing competing bacteria or become inactive during the period of storage.

Similarly, Tao et al. [44], in a study conducted on fish samples from Fiji also found low histamine levels. They argued that although the fish samples under study did not cause histamine poisoning, the fish were a potential danger for consumption if they were kept for longer time under high temperature because histamine usually accumulate in the fish rapidly, following an increase in histamine forming bacteria. On the other hand, BT (Figure 4(b)) for DS showed significant increase in all the analyzed samples along the CCPs from CCP 1 to CCP 3. The temperature reached 30.5°C overnight during storage and auctioned periods, where high histamine levels of 45.1 mg/L with high QI score of 15 (max QI of 20) were observed. The significant increase in histamine levels of dry seasoned BT may be due to exposure of sacks filled with tuna to the scorching sun for more than 3 hours upon arrival at the landing site, without the use of ice. The data confirmed the fact that storage temperature along the supply chain is one key principal controlling factor in histamine build-up, which Middlebrooks et al. [45] and Torido et al. [46] also observed.

It appears that histamine levels increased as tuna was transferred from CCP 1 to CCP 3 in both fresh and brined tuna for both seasons (Figures 4(a) and 4(b)). However, fortunately, the histamine level seemed to still fall with the EU acceptable limit of 100 mg/kg (100 mg/L) [29]. The results confirmed strong positive correlations between TT and histamine build-up (*r* = 0.91), QI and TT (*r* = 0.80), TT and microbe (*r* = 0.70). These results demonstrate the importance of TT in retaining the freshness of fish while lowering the microbial levels in order to increase the shelf life of fish. Other parameters that showed positive correlation to histamine levels are QI (*r* = 0.85) and microbes (*r* = 0.62). These relationships may be significant in the determination of the income for fishermen or retailers when fish is purchased by consumers. Better-quality fish fetches higher price, which contributes to improve income [47].

### 4.4. Microbial Load of Tuna Flesh

Increase in total microbial count is an indicator of spoilage due to poor handling practices [48]. Although freshly caught tuna has minimal spoilage bacteria upon catch, proper postharvest handling practices with continuous cold chain along the supply chain are essential in maintaining higher quality, if fishers and fish retailers are to obtain premium price in the market [49, 50].

In this study, DS tuna showed increasing trends of microbes compared to WS for both fresh and brined tuna flesh samples. Microbe levels for FT appear to also increase along the supply chain from CCP 1 (harvest) to CCP 2 (auction site) with total colony counts of 2.6 cfu/mL and 3.7 cfu/mL, respectively, while the wet season BT has total colony counts for CCP 1 at 4.8 cfu/mL and 6.6 cfu/mL at CCP 3. Several studies by Hobbs [51], Feldhusen [52], Painter et al. [53], and Samakupa [54] argued that even though the flesh of fish is sterile before death due to its strong immune system that prevents bacteria from multiplying easily, after death, the fish immune system breaks down, allowing microorganisms to enter and cause spoilage to the fish. It is also worth noting that the total colony counts for FT on both seasons were within the acceptable limits of 10^7^ cfu/ml as per the ICSMF [55] standards. The result appears to show that the increased in TT as shown in Figures 3 and 4 caused an increase in the total microbial levels (Figure 5) for fresh and brined tuna that were sampled during both seasons. These are demonstrated in the positive correlations between TT and microbial level (*r* = 70), histamine and microbial level (*r* = 0.62), and QI and microbial level (*r* = 0.60) shown in Table 1.

#### 4.4.1. Microbial Load of Tuna Contact Surfaces

The increasing trend in the growth of microorganisms on tuna contact surfaces from morning to afternoon (Figure 6) indicates poor handling practices, especially the poor hygiene practices related to the cleaning protocols during the auction period and the noncompliance of not using ice continuously along the cold supply chain. Microbial quality of fish contact surfaces reflects the actual status quo in the cleanliness of the equipment used and the personal hygiene practices of fish handlers [56]. It was observed that the contact surfaces of unsold fish that were returned home for storage overnight were exposed to higher bacterial counts. It was also alarming to observe that the morning microbial data for the contact surfaces of DS were high. This may be due to contact surfaces not cleaned at all or not cleaned properly after the previous day's auction prior for use the following day. These data appeared to support the statement made by Adebayo-Tayo et al. [57] who argued that fish contamination is also linked to raw material, personnel, and processing tools which were observed in the current study. Improper sanitary conditions along the supply chain from primary production to consumers with the occurrence of various food borne diseases do also pose vulnerability in the safety of seafood in developing countries, like the Solomon Islands [58].

## 5. Conclusions

This study reveals the prevalence of poor handling practices along the supply chain, especially related to fish catch load, specifically during dry seasons when the catch is high. Both artisanal fishers and fish retailers have limited chilled-related resources to deal with high catch volume, leading to poor ratio of ice to fish. Evidence of poor handling practices was seen in the detection of histamine and the microbial load in tuna; however, the levels of histamine were within the European Union (EU) acceptable limit of 100 mg/kg (100 mg/L) and microbial loads in this present study did not exceed ICSMF [55] limit of 10^7^ cfu/mL. This implies that both fresh and brined tuna sold at Honiara Fish Market are not totally unsafe for consumption; however, care in the postharvest good handling practices need to be exercised particularly during high catch volume season.

Hence, in order to retain higher quality postharvest handling practices of tuna and other rich seafood in the Solomon Islands or other developing countries facing the same issue, the following key recommendations need to be adhered to closely by the government authorities:

- Provide postharvest trainings to fish handlers to improve some of the current handling practices. This may contribute to the sale of higher-quality tuna which may also attract higher-end markets
- Upgrade fish market facilities to provide vendors with adequate infrastructures and storage facilitates with clear cleaning protocols to safely store and sell fish
- Implement temperature monitoring to fish handlers along the supply chain and easier access to appropriate ice types at a cheaper rate especially at locations where there are large catches

## Figures and Tables

**Table 1:** Correlations between time-temperature, quality index, histamine, and microbe.

	TT	QI	Histamine	Microbe (tuna flesh)
TT	1	0.80	0.91	0.70
QI	—	1	0.85	0.60
Histamine	—	—	1	0.62
Microbe	—	—	—	1

## Data Availability

The data used to support the study's findings are supplied in the paper.

## References

[1] FAO (2022). Fishery and aquaculture country profiles. Solomon Islands. Country profile fact sheets. https://www.fao.org/fishery/en/facp/25/en.

[2] Doyle M. P., Beauchat L. R., Thomas J. (1997). *Food Microbiology: Fundamental Frontiers*.

[3] Delgado C. L., Wada N., Rosegrant M. W., Meijer S., Ahmed M. (2003). *Fish to 2020: Supply and Demand in Chainging Global Markets*.

[4] Garcia-Tapia G., Barba-Quintero G., Gallegos-Infante J. A., Arguilar R. P., Ruiz-Cortes A., Ramirez J. A. (2013). Influence of physical damage and freezing on histamine concentration and microbiological quality of yellowfin tuna during processing. *Food Science and Technology*.

[5] Roy B. C., Miyake M., Ando K., Kawasaki I., Ysukamasa Y. (2010). Proximate and fatty acid compositions in different flesh cuts of cultured, cultured fasted, and wild Pacific Bluefin tuna (Thunnus orientalis). *Journal of Aquatic Food Product Technology*.

[6] Feng C., Teuber S., Gershwin M. E. (2016). Histamine (Scombroid) fish poisoning: a comprehensive review. *Clinical Reviews In Allergy & Immunology*.

[7] Lehane L., Olley J. (2000). Histamine fish poisoning revisited. *International Journal of Food Microbiology*.

[8] Kim S. H., Price R. J., Morrissey M. T., Field K. G., Wei C. I., An H. J. (2002). Histamine production by morganella morganii in mackerel, albacore, Mahi-mahi, and salmon at various storage temperatures. *Journal of Food Science*.

[9] Commission Regulation (EU) No 1019/2013 of 23 October 2013 (2005). *Amending Annex I to Regulation (EC) No 2073/2005 as Regards Histamine in Fishery Products*.

[10] Commission Regulation (EU) No 231/2012 of 9 March 2012 (2012). Laying down specifications for food additives. *Listed in Annexes II and III to Regulation (EC) No 1333/2008*.

[11] Food Fraud Network EU-Coordinated Case (2017). Illegal treatment of tuna: from canning grade to sushi grade; European Commission: Brussels, Belgium, 2017. https://ec.europa.eu/food/sites/food/files/safety/docs/food-fraud_succ-coop_tuna.pdf.

[12] Muscarella M., Iammarino M., Centonze D., Palermo C. (2005). Measurement of histamine in seafood by HPLC, CE, and ELISA: comparison of three techniques. *Veterinary Research Commission*.

[13] Askar A., Reptow H. (1986). *Biogene amine*.

[14] Karovicova J., Kohajdova Z. (2005). Biogenic amines in food. *Chemical Papers*.

[15] Al-Busaidi M. A., Jukes D. J., Bose S. (2016). Seafood safety and quality: an analysis of the supply chain in the Sultanate of Oman. *Food Control*.

[16] Nielsen D., Hyldig G. N. (2004). Influence of handling procedures and biological factors on the QIM evaluation of whole herring (Clupea harengus L). *Journal of Food Research International*.

[17] Ganegama J., Jayasinghe J., Wijeyaratne M. J. S., Jayasooriya M., Hettiarachchi K. (2000). Handling practices and post-harvest losses of tuna catches from multi-day boats operating from the fish landing site Negombo, Sri Lanka. *Sri Lanka Journal of Aquatic Sciences*.

[18] Ward A., Jefferies D. J. (2000). *A manual for assessing post-harvest fisheries losses*.

[19] Kumolu-Johnson C. A., Ndimele P. E. (2011). A review on post-harvest losses in artisanal fisheries of some African countries. *Journal of Fisheries and Aquatic Science*.

[20] Ward A., Signa D. (2014). Reducing post-harvest fish losses for improved food security. SMARTFISH Programme. https://www.fao.org/3/bs226e/bs226e.pdf.

[21] FDA (1998). Scombrotoxin (histamine) formation. *Fish and Fishery Products Hazards and Control Guide*.

[22] Bremner H. A. (1985). A convenient easy to use system for estimating the quality of chilled seafood. *Fish Processing Bulletin*.

[23] Martinsdottir E., Sveindottir E., Hyldig G. (2001). *Sensory Evaluation of Fish Freshness, Reference Manual for the Fish Sector*.

[24] Sveindottir K., Hyldig G., Martinsdottir E., Jorgensen B., Kristbergsson K. (2003). Quality index method (QIM) scheme developed for farmed Atlantic salmon (Salmo salar). *Food Quality and Preferences*.

[25] Sato T., Horiuchi T., Nishimura I. (2005). Simple and rapid determination of histamine in food using a new histamine dehydrogenase from rhizobium sp. *Analytical Biochemistry*.

[26] Baranowski J. D., Frank H. A., Brust P. A., Chongsiriwatana M., Premaaratne R. J. (1990). Decomposition and histamine content in mahimahi (Coryphaena Hippurus). *Journal of Food Protein*.

[27] Chamberlain T. (2001). Histamine levels in longlined tuna in Fiji: a comparison of samples from two different body sites and the effect of storage at different temperatures. *South Pacific Journal of Natural Science*.

[28] Downes F. P., Ito K. (2015). *Compedium of Methods for the Microbiological testing of Foods*.

[29] European Parliament (2005). Commission regulation (EC) no 2073/2005- microbiological criteria for foodstuffs. *Official Journal of the European Union*.

[30] Kumar R. (2011). *Research Methodology. A Step-by-Step Guide for Beginners (Vol. 3)*.

[31] Lawless H. T., Heymann H. (1998). *Sensory Evaluation of Food: Principles and Practices*.

[32] Guizani N., Al-Busaidi M. A., Al-Belushi I. M., Mothershaw A., Rahman M. S. (2005). The effect of storage temperature on histamine production and the freshness of yellowfin tuna (Thunnus albacares). *Food Research International*.

[33] Akintola S. L., Bakare S. B. (2011). Microbiological changes in freshwater prawn (Macrobrachium vollenhovenii, Herklots 1857) stored in ice. *American Journal of Food Technology*.

[34] Burns F. D. (1985). Tuna handling and refrigeration on purse seiners. Carlifonia: U S Department of Commerce. http://www.seafood.oregonstate.edu/..../Tuna%20Handling%20and%20Refrigeration%20on%20P.

[35] Whittle K., Hardy R., Hobbs G. (1990). Chilled fish and fishery products. *In chilled foods, the state of the art*.

[36] Olafsdottir G., Martinsdottir E., Oehlenschlager J. (1997). Methods to evaluate fish freshness in research and industry. *Trends in Food Science & Technology*.

[37] Taoukis P. S., Labuza T. P. (1989). Reliability of time and temperature indicators as food quality monitors under nonisothermal conditions. *Journal of Food Science*.

[38] Taoukis P. S., Labuza T. P., Bourgeois C. M., Roberts T. A. (1999). Chemical time-temperature intergrators as quality monitors in the chill chain. *Predictive Microbiology Applied to Chilled Food Preservation, Refrigeration Science and Technology Proceedings Series*.

[39] Huss H. H. (1995). *Quality and Quality Changes in Fresh Fish*.

[40] Shawyer M., Medina P. A. F. (2003). *The Use of Ice on Small Fishing Vessels*.

[41] Lako J., Solo M., Ishigaki M. (2016). Postharvest handling practices and the development of histamine in Giant Trevalley (Caranx ignobilis) fish the case of Fiji. *The South Pacific Journal of Natural and Applied Sciences*.

[42] Allen D. G. (2004). *Regulatory Control of Histamine Production in North Carolina Harvested Mahi-Mahi (Coryphaena Hippurus) and Yellowfin Tuna (Thunnus Albacares): A HACCP Based Industry Survey Department of Food Science, [M.S. thesis]*.

[43] Omura Y., Proce R. J., Olcott H. S. (1978). Histamine-forming bacteria isolated from spoiled tuna and jack mackerel. *Journal of Food Science*.

[44] Tao T., Sato T., Hongmei Z., Toshiyasu Y., Nakano T. (2011). A survey of histamine content in seafood sold in markets of nine countries. *Food Control*.

[45] Middlebrooks B. L., Toom P. M., Douglas W. L., Harrison R. F., McDowell S. (1988). Effects of storage time and temperature on the microflora and amine-development in Spanish mackerel (Scomberomorus maculatus). *Journal of Food Science*.

[46] Torido Y., Takahashi H., Kuda T., Kimura B. (2012). Analysis of the growth of histamine-producing bacteria and histamine accumulation in fish during storage at low temperatures. *Food Control*.

[47] Mativa G., Wakabayashi Y., Tekenouchi N. (2005). Factors influencing the prices of fish in central region of Malawi and its implications on the development of aquaculture in Malawi. *Journal of Applied Science*.

[48] Sousa C. P. (2008). The impact of food manufacturing practices on food borne diseases. *Food Science and Technology*.

[49] Lako J. (2015). *Huge Losses to Post-Harvest Handling*.

[50] Sikorski Z. E. (1990). *Seafood Resources, Nurtritional Composition and Preservation*.

[51] Hobbs G., Roberts T., Skinner F. A. (1983). Microbial spoilage of fish. *Food Microbiology: Advances and Prospects*.

[52] Feldhusen F. (2000). The role of seafood in bacterialfoodborne diseases. *Microbes Infection*.

[53] Painter J. A., Hoekstra R. M., Ayers T. (2013). Attribution of foodborne illnesses, hospitalizations, and deaths to food commodities by using outbreak data, United States, 1998-2008. *Emerging Infections Diseases Journal*.

[54] Samakupa P. (2003). *Hygiene indicators in a fish processing establishment. A case study in a white fish processing establishment*.

[55] Roberts T. A., van Schothorst M., Sharpe A. N. (1996). The International Commission on Microbiological Specification for Foods (ICMSF). *Food Control*.

[56] Jacxsens L., Kussaga J., Lunning P. A., Spiegel M. V. D., Devlieghere F., Uyttendaele M. (2009). A microbial assessment scheme to measure microbial performance of food safety management systems. *International Journal of Food Microbiology*.

[57] Adebayo-Tayo B. C., Odu N. N., Anyamele L. M., Igwiloh N., Okonko I. O. (2012). Microbial quality of frozen fish sold in Uyo Metropolis. *Nature and Science*.

[58] Sudheesh P. S., Al-Ghabshi A., Al-Abousi N., Al-Gharabi S., Al-Khadhuri H. (2013). Evaluation of food contact surface contamination and the presence of pathogenic bacteria in seafood retail outlets in the Sultanate of Oman. *Advance Journal of Food Science and Technology*.

